# A Community of One: Social Cognition and Auditory Verbal Hallucinations

**DOI:** 10.1371/journal.pbio.1001723

**Published:** 2013-12-03

**Authors:** Vaughan Bell

**Affiliations:** King's College London, London, United Kingdom

## Abstract

Auditory verbal hallucinations have attracted a great deal of scientific interest, but despite the fact that they are fundamentally a social experience—in essence, a form of hallucinated communication—current theories remain firmly rooted in an individualistic account and have largely avoided engagement with social cognition. Nevertheless, there is mounting evidence for the role of social cognitive and social neurocognitive processes in auditory verbal hallucinations, and, consequently, it is proposed that problems with the internalisation of social models may be key to the experience.

## Abstract 2

Vaughan Bell argues that because hallucinated voices are often experienced as distinct social identities we need to understand how changes in the social brain lead to these intense experiences.

## Introduction

Auditory verbal hallucinations, the experience of “hearing voices”, present us with an interesting paradox: the experiences are generated from within a single individual but are typically experienced as a social phenomenon—that is, a form of communication from another speaker.

Current theories attempt to explain auditory verbal hallucinations as alterations to individualistic information processing—namely, misattributions of internal thoughts as external phenomena due to biases in cognitive monitoring [1]. The fact that voices stem from an internal source is, of course, clear, but the typical experience of “hearing voices” is not that thoughts seem to be “spoken aloud” but that hallucinated voices have a social identity with clear interpersonal relevance [2]. In other words, voices are as much hallucinated social identities as they are hallucinated words or sounds.

Nevertheless, neurocognitive theories have largely ignored how people who “hear voices” acquire what amounts to internalised social actors. To illustrate the extent of this neglect, a recent consensus statement that described an integrated cognitive model of auditory verbal hallucinations [1] included only vague mentions of “perceptual expectations”, “top down influences”, and “emotion” to address how voices become distinguishable as social identities, without any specific suggestions for how these experiences take a social form.

This clear omission is all the more surprising given the significant advances in the field of social cognitive neuroscience. Although early theories in this field focussed largely on the development of abilities (like “theory of mind” and “mentalising”), more recent work highlights the importance of developing internalised models of social actors for both “inner” social reasoning and “live” social interaction (e.g., [3],[4]). Here it is argued that alongside the well-established difficulties with source and intention monitoring that lead to misattribution, auditory verbal hallucinations may also involve a change in the neurocognitive processes that support our internal social models of people and how they behave.

## The Social Cognition of Hearing Voices

Although, at its root, all speech is a social phenomenon, hallucinatory voices have clear interpersonal characteristics above and beyond the social basis on which they rest. First, hallucinated voices are usually experienced as having identities and making coherent communicative speech acts [5] (see example in Box 1), and, second, they are primarily experienced as social actors the hearers can relate to and interact with [6].

**Box 1. Hallucinated Social Identities**

“I mean, there are two voices—Simon and Jeremy. Simon's…um…like a demon really. He's very demonic and he…says people read my mind and they know I'm evil…. I've got a year to live, that if I don't do as I'm told the horrible horseman of the apocalypse will come and get me and kill me and Armageddon will come and the world will be destroyed. And then Jeremy—he's just a little boy, he's just full of fun, you know, he'll tell me things like—um—‘Move the food from the cupboard and put it in mum's chest of 7 drawers.’ Just stupid things like that. It's funny but it's—it is annoying really.”

—Lucy from Knudson and Coyle [33]

Phenomenological studies have reported that between 30% and 69% of people who hear voices experience them as having specific personal identities (e.g., members of the family, God, celebrities) [5],[7]–[9]. The biggest study to date reported that 31% of 199 voice hearers with psychiatric diagnosis did not experience anonymous voices, 32% experienced a mix of known and unknown voices, and only 37% experienced purely anonymous voices [5]. However, these figures are likely to be a minimum estimate of whether voices are experienced as having social identities, in terms of being able to reliably distinguish them from other voices by personal characteristics, because voices may have social identities but still be anonymous (e.g., identified only as an “unknown old woman” or “a man with a deep voice”, as in Leudar et al. [7], who name them “incognito” voices). There is evidence to support this from studies that have specifically investigated this aspect of the experience. Nayani and David [9] reported that 61% of psychiatric voice hearers knew the identity of their voices, but an additional 15% had voices that were familiar but unknown. McCarthy-Jones et al. [5] found that 70% of voice hearers reported voices that, regardless of specific identity, were “like” people they had spoken to in the past. In a qualitative study of 50 psychiatric voice hearers, Beavan [10] reported that characterising voice identity, regardless of citing a specific personal identity, was a major theme of the experience of hallucinating voices. In other words, regardless of whether a voice is associated with a personal identity in the outside world, the process of recognising it as a distinct social identity is likely to be a key experience for many voice hearers.

Similarly, numerous studies have now found that voice hearers understand their connection with the voices in terms of relationships and interact with their voices in ways that “share many properties with interpersonal relationships within the social world” [6]. Most obvious in this regard is the fact that over 80% of people who experience auditory verbal hallucinations have reported that they are able to engage in interactive conversations with their voices [7],[8]. Judgements about the identity of hallucinated voices rely on perceptual features similar to those required to judge identity when listening to the voices of other speakers, with perceived identity being an important mediator of distress [11].

With regard to the effect of social environment on voices, relationships with the hallucinated voices are experienced in terms of social power, while voice hearers' perception of the external social world is mirrored in their relationship with hallucinated voices—similarly a significant mediator of distress [6],[12]. Studies on psychiatric voice hearers have indicated that the social environment plays a role not only in the formation of hallucinations but also the maintenance of the experience [12],[13]. Although the risk of psychosis is raised after the experience of trauma in general, childhood sexual and emotional abuse—but not physical abuse—predict the presence of auditory verbal hallucinations in adulthood [14],[15], suggesting a specific link with early relationship trauma. In adult life, the death of someone in a long-term, loving relationship will commonly lead to hallucinations of that specific person, and hallucinated voices are among the most common experiences [16]. In line with phenomenological evidence that voices are typically associated with known people (either directly through identity or through resemblance [5]), it seems likely that intense emotion within a relationship is associated with both the formation and content of hallucinated voices.

These results suggest that the experience of auditory verbal hallucinations is, for most voice hearers, primarily a social one, with social environment through the lifespan having a specific effect on the presence and form of voices. The fact that most voices are perceived as having social identities with which the hearer interacts in ways verifiably similar to external social relationships suggests that voices often function as internal models of social actors.

### Auditory Verbal Hallucinations and Social Cognitive Neuroscience

The majority of studies on the cognitive neuroscience of auditory verbal hallucinations have not looked at “voices” as specifically social phenomena, but there is evidence that the neural networks involved in supporting these experiences have significant overlap with areas that play a key role in social neurocognition.

Neuroimaging studies have linked auditory hallucinations to functional and structural differences in speech and language areas—most notably the superior and middle temporal gyri and the inferior frontal gyrus (Broca's area). In addition, a wider network of non-sensory areas is implicated. These include areas typically described in the auditory verbal hallucination literature as linked to cognitive monitoring—namely, the dorsolateral prefrontal cortex, anterior cingulate, and cerebellum—and areas typically linked to emotion and affect regulation—namely, the anterior insula, hippocampal and parahippocampal regions, and the orbitofrontal cortex [17],[18]. However, these areas typically described as monitoring and emotion areas are also key components in social neurocognitive networks that make up the “social brain” [19],[20].

Of particular interest in the network commonly identified in neuroimaging studies of auditory verbal hallucinations is the temporoparietal junction, now clearly identified as having a key role in verbal working memory and social cognition [20] and, according to Saxe [21], a central role in representing others' mental states. Using electroencephalography, this area has been found to be active in the second before the onset of auditory verbal hallucinations [22], and 1-Hz repetitive transcranial magnetic stimulation (used to dampen down neural excitability) applied to this area reduces hallucination intensity [23]. In the only study that has directly stimulated the temporoparietal junction (increasing neural excitability in the area), Arzy et al. [24] induced a clear experience of social imagery in non-voice-hearing, non-psychiatric participants in the form of a “sensed presence”.

Neuroimaging studies that have directly compared the experience of auditory hallucinations to imagined inner voices have found remarkably similar brain activity, indicating that voices are not likely to be just “misidentified thoughts” but specifically “misidentified voice images”, potentially experienced as unintentional because of an altered sequence of activation in the supplementary motor area and auditory perceptual areas [25],[26]. Such voice images have been found to have dissociable neural substrates for spatial and social identity properties [27].

## Towards a Social Cognitive Approach to Voices

There is good evidence that auditory verbal hallucinations are usually experienced as social entities with which voice hearers have dynamic relationships and which are associated with specific changes to brain networks used to support social cognition. It is surprising, therefore, that cognitive and neurocognitive theories of hallucinated voices have been almost entirely individualistic in their approach.

Perhaps one exception is Fernyhough's [28] developmental theory that draws on Vygotsky's account of inner speech. According to Vygotsky [29], children learn language through interaction and dialogue with others. The dialogue format is then used, first out loud, to problem-solve (children literally talk to themselves), and later becomes internal as we learn to internalise speech as thoughts, with thoughts retaining dialogic qualities and having elements of “inner self-talk”. Fernyhough [27] uses this to explain how the “misattribution of thought” model of auditory verbal hallucinations could produce speech-like experiences. However, this account still does not explain why hallucinated voices are typically experienced as distinct social identities with which the hearer has a relationship. In essence, it still strips the social phenomenology—and, therefore, it could be argued, key aspects of social cognitive processing—from the experience.

Considering the evidence that auditory verbal hallucinations involve a misattribution of internal phenomena as external due to biases in cognitive monitoring [1], we need to be clear about the nature of the internal phenomena that are being misattributed. It would be most parsimonious to assume that these phenomena stem from our normal ability to internalise models of people we know and their voices, rather than auditory hallucinations involving a de novo generation of persistent and internally vocal social identities. Accounts including internalised models of social actors suggest that we internalise others' voices and personalities so that we can predict what someone would say or do in any given situation [30]. These internal models can be for specific people, so I can imagine how my spouse might respond in a hypothetical conversation, or for generic stereotypes, so I can imagine how a policeman or shopkeeper might respond.

Neuropsychological models of voice hearing [17],[18] suggest there is an important component involved in the generation of heard speech (changes to activity in the language system) and an important component involved in difficulty distinguishing internal from external phenomena (altered cognitive monitoring). The hypothesis suggested here is that, in addition to these well-established factors, there is an alteration to the social cognitive or social neurocognitive systems that support internal models of social actors and their associated voice imagery, to explain why voices are typically experienced as having an identity and acting socially.

It is worth examining what this implies. First, it implies that an ability to internalise models of other people and generate associated imagery of their voices is a normal developmental process. Longitudinal studies looking at developmental psychology should see that this ability develops in line with other social cognitive processes, and that people with difficulties in internalising predictive models of other individuals should have marked social difficulties. Secondly, it implies that changes to the normal functioning of this system play a causal role in many voice-hearing experiences. Although typically experienced as social, voice-hearing experiences can range from simple repetitive syllables to conversations between the hearer and several hallucinated vocal identities. Drawing on the work of Hassabis et al. [30], the more a voice is experienced as having a social identity, the more it should involve processes that support the creation of and prediction from internalised personality models at the cognitive and neurobiological level.

Furthermore, considering that one of the key experiences of voice hearing is the lack of agentive control over the voices, and that there is a link between social stress, trauma, and auditory hallucinations, the internal social models of individuals associated with intense traumatic or emotional experiences should be less predictable—and resultant imagery more intrusive—than for individuals not associated with such experiences. In addition, recent evidence has emerged that psychiatric voice hearers are much more likely to identify voices as specific living people than non-clinical voice hearers [31], suggesting that social cognitive factors may differ depending on the level of disability associated with the experience.

To further clarify these issues there is a clear need for more fundamental evidence to be gathered about the nature of voice-hearing experiences. Studies on how voices are distinguished as “individuals”, which social characteristics they are perceived to have, and how this is distinct from being associated with specific identifiable persons in the outside world (as opposed to voices with distinguishable personalities who remain “anonymous” or “incognito”) are still lacking. Furthermore, little attention has been paid to how voices evolve over time [32], and knowing when voices become “social” may be key to understanding the role of social cognition in the aetiology of auditory verbal hallucinations.

Over the last decade, psychological-level research has focussed on the link between social cognition and auditory verbal hallucinations and has amassed a significant amount of evidence as a result. However, researchers working in cognitive neuroscience, who are specifically looking to make links with neurobiology, have only occasionally engaged with studies that have investigated the social neurocognition of hearing voices. Despite some provocative results, they have not yet used paradigms that would disentangle the extent to which the “social brain” is part of the hallucinatory experience. This is clearly an area where more targeted research needs to be completed. Similarly, more effort needs to be put into developing theories that include the socially relevant evidence, as this has been largely ignored in both cognitive and neurocognitive accounts.

As one of our most enigmatic experiences, “hearing voices” is at once both individual and social. There is a clear need to understand it in terms of the individual mind and brain, and a clear opportunity for it to shed light on the social world that lives within us.

## References

[1] Waters F, Allen P, Aleman A, Fernyhough C, Woodward TS (2012) Auditory hallucinations in schizophrenia and nonschizophrenia populations: a review and integrated model of cognitive mechanisms. Schizophr Bull 38: 683–693. 22446568 10.1093/schbul/sbs045 PMC3406530

[2] Larøi F, Sommer IE, Blom JD, Fernyhough C, Ffytche DH, et al (2012) The characteristic features of auditory verbal hallucinations in clinical and nonclinical groups: state-of-the-art overview and future directions. Schizophr Bull 38: 724–733. 22499783 10.1093/schbul/sbs061 PMC3406519

[3] Brown EC, Brüne M (2012) The role of prediction in social neuroscience. Front Hum Neurosci 6: 147. 22654749 10.3389/fnhum.2012.00147 PMC3359591

[4] Gallotti M, Frith CD (2013) Social cognition in the we-mode. Trends Cogn Sci 17: 160–165. 23499335 10.1016/j.tics.2013.02.002

[5] McCarthy-Jones S, Trauer T, Mackinnon A, Sims E, Thomas N, et al (2012) A new phenomenological survey of auditory hallucinations: evidence for subtypes and implications for theory and practice. Schizophr Bull E-pub ahead of print. 10.1093/schbul/sbs156 PMC3885292 23267192

[6] Hayward M, Berry K, Ashton A (2011) Applying interpersonal theories to the understanding of and therapy for auditory hallucinations: a review of the literature and directions for further research. Clin Psychol Rev 31: 1313–1323. 21996292 10.1016/j.cpr.2011.09.001

[7] Leudar I, Thomas P, McNally D, Glinski A (1997) What voices can do with words: pragmatics of verbal hallucinations. Psychol Med 27: 885–898. 9234466 10.1017/s0033291797005138

[8] Garrett M, Silva R (2003) Auditory hallucinations, source monitoring, and the belief that “voices” are real. Schizophr Bull 29: 445–457. 14609239 10.1093/oxfordjournals.schbul.a007018

[9] Nayani TH, David AS (1996) The auditory hallucination: a phenomenological survey. Psychol Med 26: 177–189. 8643757 10.1017/s003329170003381x

[10] Beavan V (2011) Towards a definition of “hearing voices”: a phenomenological approach. Psychosis 3: 63–73.

[11] Badcock JC, Chhabra S (2013) Voices to reckon with: perceptions of voice identity in clinical and non-clinical voice hearers. Front Hum Neurosci 7: 114. 23565088 10.3389/fnhum.2013.00114 PMC3615181

[12] Paulik G (2012) The role of social schema in the experience of auditory hallucinations: a systematic review and a proposal for the inclusion of social schema in a cognitive behavioural model of voice hearing. Clin Psychol Psychother 19: 459–472. 21774037 10.1002/cpp.768

[13] Goldstone E, Farhall J, Ong B (2012) Modelling the emergence of hallucinations: early acquired vulnerabilities, proximal life stressors and maladaptive psychological processes. Soc Psychiatry Psychiatr Epidemiol 47: 1367–1380. 22045103 10.1007/s00127-011-0446-9

[14] Bentall RP, Wickham S, Shevlin M, Varese F (2012) Do specific early-life adversities lead to specific symptoms of psychosis? A study from the 2007 the Adult Psychiatric Morbidity Survey. Schizophr Bull 38: 734–740. 22496540 10.1093/schbul/sbs049 PMC3406525

[15] Daalman K, Diederen KM, Derks EM, van Lutterveld R, Kahn RS, et al (2012) Childhood trauma and auditory verbal hallucinations. Psychol Med 42: 2475–2484. 22716897 10.1017/S0033291712000761

[16] Keen C, Murray C, Payne S (2013) Sensing the presence of the deceased: a narrative review. Ment Health Relig Cult 16: 384–402.

[17] Allen P, Modinos G, Hubl D, Shields G, Cachia A, et al (2012) Neuroimaging auditory hallucinations in schizophrenia: from neuroanatomy to neurochemistry and beyond. Schizophr Bull 38: 695–703. 22535906 10.1093/schbul/sbs066 PMC3406523

[18] Allen P, Larøi F, McGuire PK, Aleman A (2008) The hallucinating brain: a review of structural and functional neuroimaging studies of hallucinations. Neurosci Biobehav Rev 32: 175–191. 17884165 10.1016/j.neubiorev.2007.07.012

[19] Kennedy DP, Adolphs R (2012) The social brain in psychiatric and neurological disorders. Trends Cogn Sci 16: 559–572. 23047070 10.1016/j.tics.2012.09.006 PMC3606817

[20] Rushworth MF, Mars RB, Sallet J (2013) Are there specialized circuits for social cognition and are they unique to humans? Curr Opin Neurobiol 23: 436–442. 23290767 10.1016/j.conb.2012.11.013

[21] Saxe R (2006) Uniquely human social cognition. Curr Opin Neurobiol 16: 235–239. 16546372 10.1016/j.conb.2006.03.001

[22] Line P, Silberstein RB, Wright JJ, Copolov DL (1998) Steady state visually evoked potential correlates of auditory hallucinations in schizophrenia. Neuroimage 8: 370–376. 9811555 10.1006/nimg.1998.0378

[23] Slotema CW, Aleman A, Daskalakis ZJ, Sommer IE (2012) Meta-analysis of repetitive transcranial magnetic stimulation in the treatment of auditory verbal hallucinations: update and effects after one month. Schizophr Res 142: 40–45. 23031191 10.1016/j.schres.2012.08.025

[24] Arzy S, Seeck M, Ortigue S, Spinelli L, Blanke O (2006) Induction of an illusory shadow person. Nature 443: 287. 16988702 10.1038/443287a

[25] Linden DE, Thornton K, Kuswanto CN, Johnston SJ, van de Ven V, et al (2011) The brain's voices: comparing nonclinical auditory hallucinations and imagery. Cereb Cortex 21: 330–337. 20530219 10.1093/cercor/bhq097

[26] Shergill SS, Bullmore E, Simmons A, Murray R, McGuire P (2000) Functional anatomy of auditory verbal imagery in schizophrenic patients with auditory hallucinations. Am J Psychiatry 157: 1691–1693. 11007729 10.1176/appi.ajp.157.10.1691

[27] Rämä P, Poremba A, Sala JB, Yee L, Malloy M, et al (2004) Dissociable functional cortical topographies for working memory maintenance of voice identity and location. Cereb Cortex 14: 768–780. 15084491 10.1093/cercor/bhh037

[28] Fernyhough C (2004) Alien voices and inner dialogue: towards a developmental account of auditory verbal hallucinations. New Ideas Psychol 22: 49–68.

[29] Vygotsky L (1934) Thought and language. Cambridge (Massachusetts): MIT Press.

[30] Hassabis D, Spreng RN, Rusu AA, Robbins CA, Mar RA, et al (2013) Imagine all the people: how the brain creates and uses personality models to predict behavior. Cereb Cortex E-pub ahead of print. 10.1093/cercor/bht042 PMC4089378 23463340

[31] Daalman K, van Zandvoort M, Bootsman F, Boks M, Kahn R, et al (2011) Auditory verbal hallucinations and cognitive functioning in healthy individuals. Schizophr Res 132: 203–207. 21839618 10.1016/j.schres.2011.07.013

[32] Hartigan N, McCarthy-Jones S, Hayward M (2013) Hear today, not gone tomorrow? An exploratory longitudinal study of auditory verbal hallucinations (hearing voices). Behav Cogn Psychother 19: 1–7. 10.1017/S1352465813000611 23866079

[33] Knudson B, Coyle A (2002) The experience of hearing voices: an interpretative phenomenological analysis. Existent Anal 13: 117–134.

